# Health of Louisville

**Published:** 1840-10

**Authors:** 


### HEALTH OF LOUISVILLE.

From the unusual quantity of rain which fell in this city in the last Spring, and first Summer months, it was apprehended that the Fall would be sickly. Up to this time, we are happy to say, these apprehensions have not been realized. The cases of Fever have not been numerous, and, generally, those which have presented have been of an uncommonly mild and manageable character. Upon the whole, it is the opinion of the oldest physicians of the city that few more healthy seasons have passed over Louisville. Y.

